# Surface Area of Graphene Governs Its Neurotoxicity

**DOI:** 10.1021/acsbiomaterials.3c00104

**Published:** 2023-05-18

**Authors:** Şeyma Taşdemir, Zehra Gül Morçimen, Aslı Aybike Doğan, Cansu Görgün, Aylin Şendemir

**Affiliations:** † Bioengineering Department, Celal Bayar University, Manisa 45140, Turkey; ‡ Department of Bioengineering, Ege University, Izmir 35040, Turkey; ∥ Department of Experimental Medicine (DIMES), University of Genova, Genova 16126, Italy; ⊥ Department of Biomedical Technologies, Ege University, Izmir 35040, Turkey

**Keywords:** graphene, neurotoxicity, cytotoxicity, genotoxicity, SH-SY5Y

## Abstract

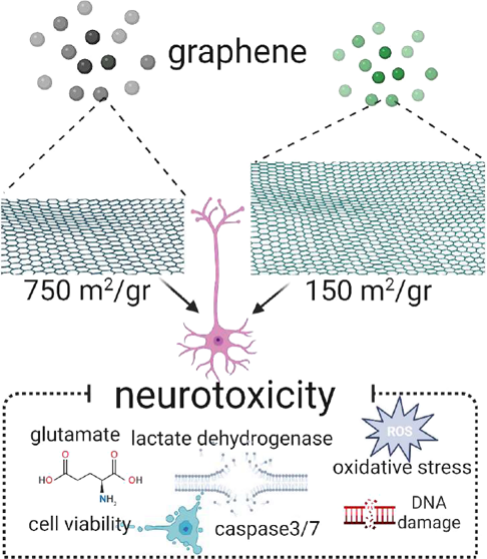

Due to their unique physicochemical properties, graphene and its derivatives are widely exploited for biomedical applications. It has been shown that graphene may exert different degrees of toxicity in in vivo or in vitro models when administered via different routes and penetrated through physiological barriers, subsequently being distributed within tissues or located within cells. In this study, in vitro neurotoxicity of graphene with different surface areas (150 and 750 m^2^/g) was examined on dopaminergic neuron model cells. SH-SY5Y cells were treated with graphene possessing two different surface areas (150 and 750 m^2^/g) in different concentrations between 400 and 3.125 μg/mL, and the cytotoxic and genotoxic effects were investigated. Both sizes of graphene have shown increased cell viability in decreasing concentrations. Cell damage increased with higher surface area. Lactate dehydrogenase (LDH) results have concluded that the viability loss of the cells is not through membrane damage. Neither of the two graphene types showed damage through lipid peroxidation (MDA) oxidative stress pathway. Glutathione (GSH) values increased within the first 24 and 48 h for both types of graphene. This increase suggests that graphene has an antioxidant effect on the SH-SY5Y model neurons. Comet analysis shows that graphene does not show genotoxicity on either surface area. Although there are many studies on graphene and its derivatives on their use with different cells in the literature, there are conflicting results in these studies, and most of the literature is focused on graphene oxide. Among these studies, no study examining the effect of graphene surface areas on the cell was found. Our study contributes to the literature in terms of examining the cytotoxic and genotoxic behavior of graphene with different surface areas.

## 1 Introduction

Carbon is a nonmetallic element in the 4A group of the periodic table, and it is very common in nature. Carbon sp, sp2, and sp3 hybridizations allow for the formation of a large number of carbon allotropes, such as amorphous carbon, graphite, diamond, and fullerene. Graphene is known as a nanomaterial with superior properties due to the fact that it is two-dimensional (2D), and covalently bonded carbon atoms are perfectly aligned in a hexagonal honeycomb mesh.^1−3^ This material was discovered in 2004 by Novoselov and his team demonstrating that they can synthesize a single layer of graphene and have much more varied electronic and physical properties than expected.^4^ The “ground-breaking experiments’’ about graphene brought Dutch physicist Andre Geim and Russian-born British citizen Konstantin Novoselov the Nobel Prize in Physics in 2010.^4^

The electrons in graphene act as massless relativistic particles at room temperature. Thus, graphene exhibits unique properties, such as quantum void effect. The main characteristics of the graphene are its high surface area (up to 2600 m^2^/g), high electron mobility (10000 cm^2^/Vs at room temperature), and resulting high electrical (∼4000 Wm^–1^/K) and thermal conductivity (5000 Wm^–1^/K), high Young’s modulus (1000 GPa), and high fracture strength (130 GPa).^5,6^ Due to these unique properties, its use in biomedical applications, such as drug and gene delivery systems, phototherapy applications, and tissue engineering studies, has become important.^7−10^ Its high surface area, nanoroughness, and electrical conductivity make graphene particularly a good candidate for neural tissue engineering and biomaterial-based neuroregenerative therapies, where traditional materials are insufficient.

The specific surface area of a specific nanoparticle mass is greater than the specific surface area of the microparticles at the same amount. Increased surface area and surface area/volume ratio provide more surface interaction. When used in biological environments, this large surface area increases the adsorption of proteins and other biomolecules on the surface. In addition, materials with nanoroughness show more similarity to natural tissue than traditional materials. Cellular interaction with a nanoscale biomaterial provides physiologically active cell surface receptors to increase interactions and provide further regeneration.^11,12^ Thus, these materials become a good substrate for tissue regeneration.

Appropriate electrical conductivity is very important for the responses of neural cells. It is thought that electrical stimulation is necessary for the regeneration of neural tissue in both the central nervous system (CNS) and peripheral nervous system (PNS).^13^

Unlike carbon nanoparticles or nanotubes, much less is known about the interactions of graphene nanoparticles with target cells and their potential toxicity. The interaction between layers of graphene or graphene oxide (GO) and target cells was studied in cultures of lung epithelial cells, fibroblasts, macrophages, breast cancer cells, stem cells, and neuronal cells.^14−18^ Although some of these studies presented findings on the toxicity of graphene, some reported that no cytotoxic effect was observed. In a study by Chang et al. in 2011 with human lung epithelial A549 cells, it was found that at high concentrations of GO, dose-dependent oxidative stress increased and cell viability decreased.^19^ On the contrary, in their study with A549 cells, Chong et al. reported that graphene quantum dots did not have significant cytotoxicity.^20^ It seems graphene-based nanomaterials can be either toxic or biocompatible to cells depending on the characteristics of graphene or the cell type. Data on graphene cytotoxicity are conflicting, and varying physical properties of graphene affect the results. Therefore, the mechanisms of toxicity and influencing factors need to be investigated in detail.

Nerve injuries and neurodegenerative diseases lead to neurite damage and neuronal loss. There are many biomaterial-based regenerative studies that are being developed to repair damaged neurons.^21−23^ Recently, the use of graphene-based tissue scaffolds in neuroregeneration has become important. The electroactive and conformational properties of graphene stimulate and guide neural cells and increase neural proliferation and differentiation.^24,25^ However, most conductive materials are toxic and prone to biodegradation. GO nanoparticles,^24,26−29^ 2D graphene films,^30−32^ and three-dimensional (3D) graphene scaffolds^33,34^ have been found promising in the regeneration of nerve cells. However, graphene scaffolds are not biodegradable and the effects of graphene on neurons remain unclear. In order to increase the in vivo and clinical applications of graphene, graphene–cell interactions need to be better known and controlled.

The ISO 19007:2018 standard has been published on the cytotoxic investigation of nanoparticles. The standard was used in cytotoxicity analysis; however, no arguments on genotoxicity are made in this standard. In our study, the cytotoxic and genotoxic effects of graphene on neurons depending on the surface area were investigated.

## 2 Materials and Methods

### 2.1 Cell Culture

The human neuroblastoma cell line, SH-SY5Y (CRL-2266TM, ATCC), was obtained from the Ege Biomaterials and 3D Biointerphases laboratories collection, Bioengineering Department of Ege University. SH-SY5Y cells, one of the most frequently used cell lines in neuroscience studies, are human-derived neuroblastoma cells that secrete catecholamines, such as dopamine, and exhibit dopaminergic neuron-like properties, possess ion channels, and neurotransmitter receptors.^35−39^ No chemical induction was used in 81.5% of studies in which SH-SY5Y cells were used as neuronal model cells in the literature.^40^ These cells provide great advantages because it is easier to obtain these cells than primary cells,^36^ to preserve their structural stability after genetic manipulations,^41^ and to create toxicity-induced disease models and to use these models in drug trials. Cells were cultured in Dulbecco’s modified Eagle’s medium, high-glucose (DMEM-HG; F0445; Biochrom) media containing 10% (v/v) fetal bovine serum (FBS; A0500-3010; CellProGen) and 1% l-glutamine (K0282; Biochrom) and incubated in a humidified incubator at 37 °C and containing 5% CO_2_. Cells between passages 10 and 15 were used. The media were changed every other day, and cells reaching 80% confluency were passaged.

### 2.2 SH-SY5Y Cells’ Exposure to Graphene

SH-SY5Y cells were plated onto different culture plates for different tests described in Table 1 and incubated for 24 h in a humidified incubator at 37 °C containing 5% CO_2_. After 24 h of incubation, graphene (Nanografi Nanotechnology, Turkey) with two different surface areas (150 and 750 m^2^/g) was sterilized by ultraviolet (UV) light and was added to the culture media at different concentrations (3.125–400 μg/mL graphene). Cells were incubated for 24–72 h with graphene before each test was conducted.

**Table 1:** Physical Properties of Graphene Powders Provided by the Manufacturer

powder	surface area (m^2^/g)	bulk density (g/cm^3^)
graphene 1	150	0.05
graphene 2	750	0.2–0.4

### 2.3 Cell Viability

Cells were seeded onto 96-well culture plates at a concentration of 5 × 10^4^ cells/mL (*n* = 3), and at the end of 24–72 h periods, cell viability was measured by the 3-(4,5-dimethylthiazol-2-yl)-2,5-diphenyltetrazolium bromide (MTT; M5655; Sigma) test.^42^ The used medium was replaced by the serum-free medium containing 10% of MTT (5 mg/mL). Cells were incubated in the dark at 37 °C for 3 h in a 5% CO_2_ incubator. After 3 h of incubation, medium containing MTT was removed. Dimethyl sulfoxide (DMSO; 41646; Sigma) was added to the cells to dissolve the formed formazan crystals, and the absorbance values were recorded at 570 nm by the ELISA reader (ELx800UV; BioTek).

### 2.4 LDH

LDH analysis is an assay used to determine the toxicity of nanoparticles.^43^ Only a high (400 μg/mL), a medium (25 μg/mL), and a low (3.125 μg/mL) concentration of graphene were tested for LDH levels (4744926001; Roche). Cells were seeded onto 96-well culture plates at a concentration of 5 × 10^4^ cells/mL (*n* = 3). After 24, 48, and 72 h, the plate was centrifuged at 250*g* for 10 min to completely settle the cells and the particles present in the medium, thus allowing measurement from a clean supernatant. Then, a 10 μL of the supernatant medium from each well was transferred to a separate plate. A total of 90 μL serum-free medium was added to 10 μL samples to reduce the total serum ratio to 1%. 100 μL reaction mixture was added to each well. The cells were incubated with the reaction mixture for 30 min in the incubator. In this step, a red color change was observed directly linked to the amount of LDH. At the end of 30 min, 50 μL of stop solution was added. In the ELISA reader, absorbance values at the wavelength of 490 nm were recorded. High control was obtained by adding lysis buffer to the cultured cells as recommended by the kit, and it determines maximum LDH release. Low control was obtained by mixing healthy cells and serum-free medium, and it determines spontaneous LDH release. Cytotoxicity was calculated according to the following equation:
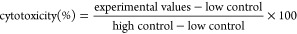


### 2.5 MDA

Only the highest (400 μg/mL) and lowest (3.125 μg/mL) concentrations of graphene were tested for MDA levels (MAK085; Sigma).^44^ Cells were seeded onto six-well culture plates at a concentration of 4 × 10^5^ cells/mL (*n* = 3). After 24, 48, and 72 h, culture plates were placed on ice, the media in the wells were withdrawn, and the cells were lysed by a lysis buffer and butyl hydroxy toluene (BHT). Subsequently, the cells were centrifuged at 13,000*g* for 10 min. After centrifugation, 200 μL of the supernatant was transferred from the tubes to prepare the sample tubes. Thiobarbituric acid (TBA) solution was added to each tube that contains the samples and the standards, and the tubes were incubated at 95 °C for 1 h. After the incubation, 200 μL of each tube was transferred to a 96-well culture dish. Finally, the absorbance values were determined by measuring at 532 nm wavelength in a spectrophotometer (Molecular Devices). The amount of MDA was calculated according to the following equation:
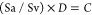
where Sa is the amount of unknown MDA in the unknown sample (nmole), according to the standard curve; Sv is the sample volume (mL) or amount (mg) added to the wells; C is the concentration of MDA in the sample; and D is the sample dilution factor (if applied).

### 2.6 GSH

The highest (400 μg/mL) and lowest (3.125 μg/mL) concentrations of graphene were tested for glutathione levels (CS0260; Sigma).^45^ Cells were seeded onto six-well culture plates at a concentration of 1 × 10^8^ cells/mL (*n* = 3). The samples were centrifuged at 600*g*, and the supernatant was removed. 5% 5-Sulfosalicylic acid (SSA) was added to deproteinize the samples. GSH causes the continuous reduction of 5,5-dithiobis(2-nitrobenzoic acid) (DTNB) to TNB, and the formed GSSG is recycled by glutathione reductase and NADPH. As a result of the kinetic reaction in this conversion, the GSSG reacts to give a positive value. The reaction rate is proportional to the glutathione concentration up to 2 μM. 5-Thio2-nitrobenzoic acid (TNB) is measured spectrophotometrically at 412 nm (BioTek Synergy HTX).

### 2.7 Caspase 3/7

Caspase 3/7 analysis is used to detect DNA fragmentation causing apoptosis.^46^ Cells were seeded onto 96-well culture plates at a concentration of 5 × 10^5^ cells/mL (*n* = 3). Cells were removed from the surface by standard trypsinization procedure and transferred to Eppendorf tubes in media. Cells were centrifuged at 1000 rpm, and the supernatant was removed. The buffer solution was added onto the pellet and left on ice for 10 min. At the end of the incubation on ice, the cells were centrifuged for 1 min at 10 ,000 rpm. A 100 μL supernatant was transferred to a 96-well plate, and dithiothreitol (DTT) and buffer solution were added onto the supernatant and incubated for 2 h in the dark in a shaker at 300 rpm at 37 °C. At the end of the incubation time, the plate was read at 405 nm in an ELISA reader (ELx800UV; BioTek). 2 μM staurosporine (S6942; Sigma) was used as a positive control to determine whether the Caspase 3/7 (GR107005; Genorise) test was working correctly. The negative control caspase activation rate was accepted as 100, and other groups were calculated accordingly.

### 2.8 Genotoxicity

OxiSelect Comet test kit (STA-351) was used for genotoxicity. Measurements were carried out with Cleaver Scientific, COMPAC-50 device. Three different positive controls were used. The first positive control was doxorubicin, a chemical that damages DNA, causing disruption of cellular mitochondria, inhibition of cell proliferation, and cell death.^47^ The second positive control was 6-hydroxydopamine, a neurotoxin that induces apoptosis in neurons. Cells were treated with 100 μM 6-OHDA for 24 h,^48^ and the third positive control was ultraviolent (UV) exposure. Prolonged UV exposure causes cellular DNA damage and induction of apoptosis.^49^ Cells were exposed to UV (362 nm) for 1 h. Healthy cells were used as the negative control.

A 10 μL of cell suspension was mixed with 100 μL of low-boiling grade (LMP) agar contained in the kit. The prepared cell–gel mixture was added onto slides on a completely flat and cold (+4 °C) surface without creating air bubbles. The gels were allowed to be set for 15 min. After the gels were frozen, the slides were placed in a dark box and cold lysis solution (pH: 10) was added. Slides were kept at +4 °C for 2 h. Since the DNA released during the lysis process is sensitive to light, the processes were always carried out in cold and dark to prevent process-related breaks in the DNA. Slides were kept in an alkaline electrophoresis buffer (pH > 13) for 30 min before electrophoresis to make the free DNA into a single chain. Freshly prepared cold electrophoresis buffer of 1–2 mm exceeding the gel was added to the slides. DNA migration was achieved at 21 V, 700 mA, 150 W, and +4 °C with a short run time of 30 min. After the electrophoresis step, the slides were washed three times with distilled water at 4 °C for 2 min. After neutralization, each slide was washed by 70% ethanol for 5 min and allowed to dry. Finally, slides were stained with Vistagreen dye diluted 1/10, 000 in 1× Tris-EDTA buffer. After staining, the slides were dried for 30 min and photographed.

### 2.9 Statistical Analysis

GraphPad Prism program (version 6; GraphPad Software, Inc., San Diego, CA, USA) was used to evaluate the statistical significance of the data obtained. The statistical differences between the experimental groups were assessed by two-way ANOVA (analysis of variance) at 95% confidence interval (*p* ≤ 0.05), and the bilateral relations between the groups were determined by Tukey’s multiple comparison (post hoc) method.

## 3 Results and Discussion

### 3.1 Cell Viability

**Figure 1:**
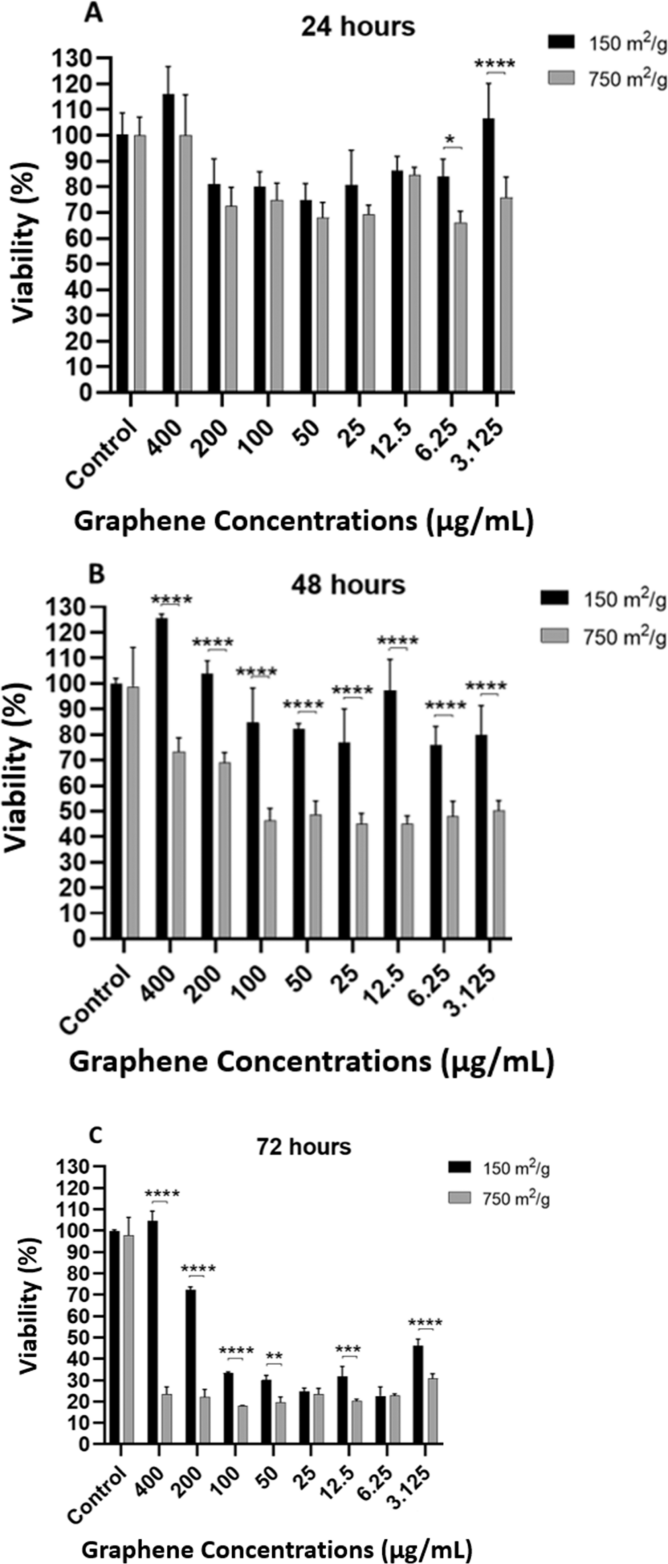
Cell viability (%) after (A) 24, (B) 48, and (C) 72 h of exposure to 150 and 750 m^2^/g graphene. Toxicity was observed for 750 m^2^/g for all three days and every graphene concentration tested (*p < 0.5, **p ≤ 0.01, ***p 0.001, ****p ≤ 0.0001).

Graphene with a surface area of 750 m^2^/g showed higher toxicity than the one with a surface area of 150 m^2^/g at 48 and 72 h (Figure 1).

Cell response depends on the purity, number of layers, size, application concentration, surface chemistry, and hydrophilicity of graphene. It has been discussed and shown by many researchers that graphene-based nanomaterials can be biocompatible or toxic to cells.^50^ There are many conflicting studies with different results despite using the same type of cell and graphene.^19,20^ In a study that had similar results with our study,^51^ the effect of GO surface area on cell viability was examined, and it was reported that large GO particles reduced cell viability compared to small ones. They correlated the decrease in viability with the time and concentration of GO exposed to cells and stated that the toxic effect increased as the time and concentration increased in HeLa Kyoto and J7442 macrophages. Researchers have stated that GO may be responsible for the biological signal that triggers increased vacuole formation, but there is no relevant finding in our study. Some studies have found that GO is in contact with the cell membrane in neurons exposed to GO, impairing autophagy and calcium homeostasis. This shows the effect of GO layers to damage neuronal transmission and functionality without causing toxicity.^52^ In studies using SH-SY5Y cells, it has been shown that GO does not induce apoptosis and has no significant cytotoxicity at low concentrations (<80 μg/mL), but the viability of cells exhibits dose and time-dependent decreses at high concentrations (≥80 μg/mL).^53^ In another study with graphene quantum dots, they have been shown to have a protective effect on SHSY5Y cells.^54^ However, in these studies, no relationship was established with the surface area of graphene.

Since there were no significant differences in cell viability between the concentrations, three graphene concentrations (400, 25, and 3.125 μg/mL) were selected and other analyses were performed with these three concentrations.

### 3.2 LDH

The LDH assay measures the cytotoxic effect of a substance given to the cells. LDH is present in the cell cytoplasm and normally is not found in the cell culture medium. If the cytotoxic substance causes damage to the cell membrane, the LDH present in the cytoplasm passes through the cell membrane to the medium, reacts with the kit, and the absorbance values increase.^55^

Particles smaller than 100 nm can pass through the cell membrane, those smaller than 40 nm can enter the cell nucleus, and those smaller than 35 nm can cross the blood–brain barrier.^56^

**Figure 2:**
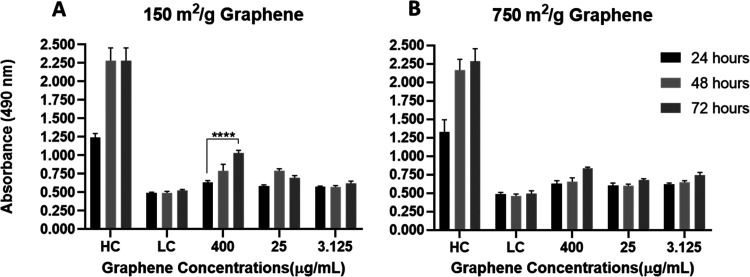
LDH-induced cytotoxicity level of (A) 150 and (B) 750 m^2^/g graphene. There is an increase in LDH for 150 m^2^/g graphene for 400 μg/mL but no significant change for other groups (****p ≤ 0.0001) (HC: high control (treated with lysis buffer), LC: low control (culture medium)).

When the LDH results are examined, no toxicity is observed, except for 400 μg/mL 150 m^2^/g sample (Figure 2). It is concluded that either type of graphene used in our study does not cause membrane damage.

Although the toxicity of graphene has been studied, the mechanisms underlying its toxicity remain unclear. Possible mechanisms of graphene cytotoxicity are considered as physical damage, oxidative stress, mitochondrial and DNA damage, apoptosis, and necrosis. The possible routes that graphene could enter the cell are via endosomes, phagosomes, or by damaging the cell membrane that could thereby affect cell metabolism. It can also cause ROS formation, LDH, and MDA increase that will damage the cell by inducing inflammatory receptors or cell receptors such as TLR. Zhang et al. reported in their study in 2010 that several layers of graphene induce cytotoxic effects on rat pheochromocytoma (PC-12) cells, and these effects depend on concentration and shape.^57^ The study reported that graphene layers increased LDH release, intracellular ROS production, and Caspase3 activation and induced apoptosis due to mitochondrial damage in neuronal cells.^57^ When Figure 2 is examined, there is no significant increase in exocellular LDH values except for the highest concentration of 150 m^2^/g graphene, and only after 72 h. It can be concluded that the toxicity measured in MTT tests is not due to membrane damage.

The change in cell viability as the concentration changes has been reported many times in different publications. Similar to our results, Chowdhury et al. showed a dose-dependent and time-dependent decrease in cell viability for HeLa, NIH-3T3, SKBR3, and MCF7 cell lines.^58^ Our LDH results show that the decrease in cell viability is not caused by membrane damage. The surface charge and surface chemistry of graphene influence the interactions between graphene and the lipids of the cell membrane.^59^ However, nanomaterials such as graphene can enter the cytoplasm without damaging the membrane with their small size and sharp edges. This, in turn, may induce toxicity by reducing the mitochondrial membrane potential within the cell, causing mitochondrial dysfunction. In addition, in a study conducted with HEK-293T cells, it was reported that in the application of small and large sizes of G and GO, the production of reactive oxygen species in the cells increased and DNA damage occurred due to this.^60^ Since Jia et al. did not report the size of L-graphene, a comparison could not be made; however, in our study, the increase in LDH ratio in 150 m^2^/g with higher concentration and larger particle size shows similar results.^60^

Liao et al. pointed out that GOs have sharp edges, so their suspensions can penetrate the cell membrane and reach the cytoplasm and cause the formation of ROS species, leading to cell death. Liao et al., who suggested that GOs be coated with a polymeric matrix to modify their sharp structure, stated that damage to the cell membrane from GO penetration could be prevented.^50,61^

### 3.3 Oxidative Stress

**Figure 3:**
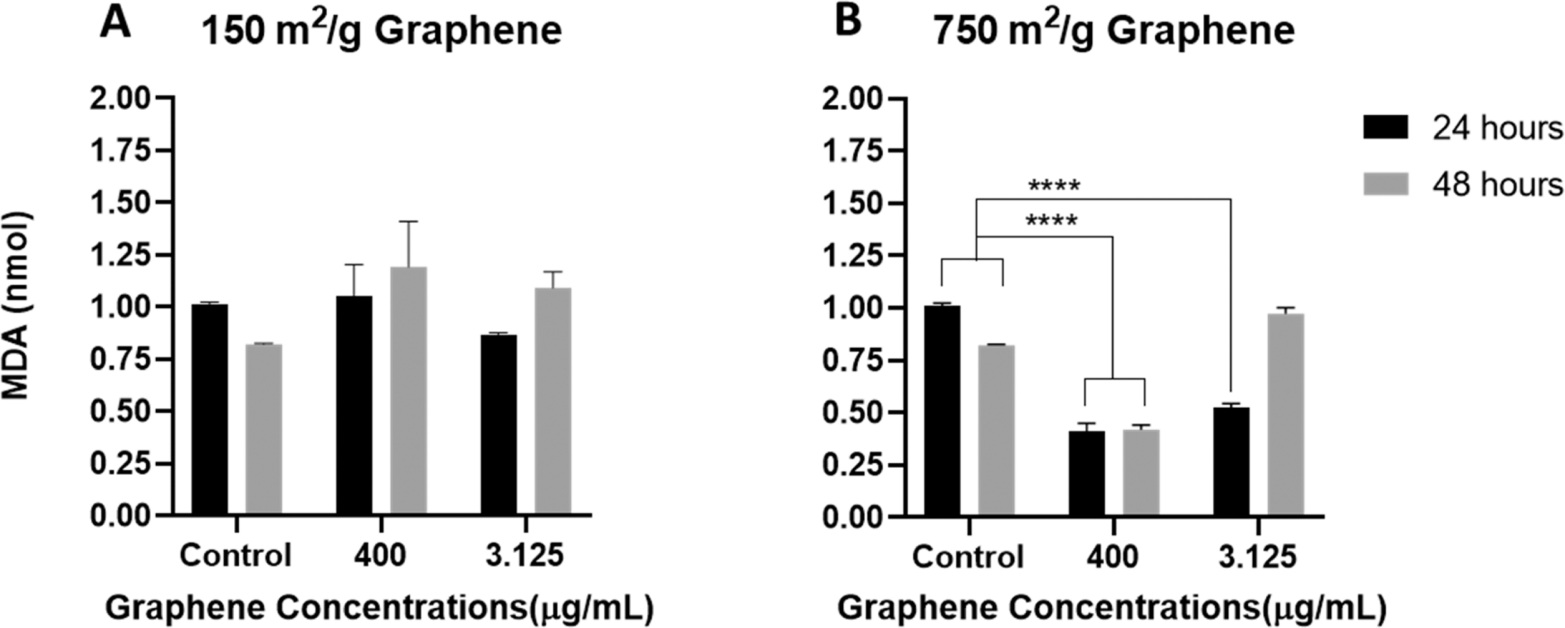
Effects of (A) 150 and (B) 750 m^2^/g graphene on MDA in SH-SY5Y cells. No significant increase in MDA was observed at any time or concentration compared to control for 150 m^2^/g graphene. (*P* > 0.05) A significant decrease in MDA was observed at the concentration of 400 μg/mL after 24 and 48 h compared to cell control (**p ≤ 0.01, ***p 0.001).

Lipid peroxidation, a marker of reactive oxygen species, is the degradation of lipids that occurs as a result of oxidative damage and indicates oxidative stress. Polyunsaturated lipids react with reactive oxygen species to form malondialdehyde (MDA). Lipid peroxidation by reactive oxygen species is known to be involved in the damaging mechanism of various acute and chronic brain disorders.^62,63^ When the results of the MDA test were examined, it was observed that, at the concentrations tested, neither of the graphenes induced oxidative stress through this pathway (Figure 3). It has been determined that the graphene with the 750 m^2^/g surface area shows less MDA levels than the cell control, implicating that this graphene type may even show an antioxidant effect.

**Figure 4:**
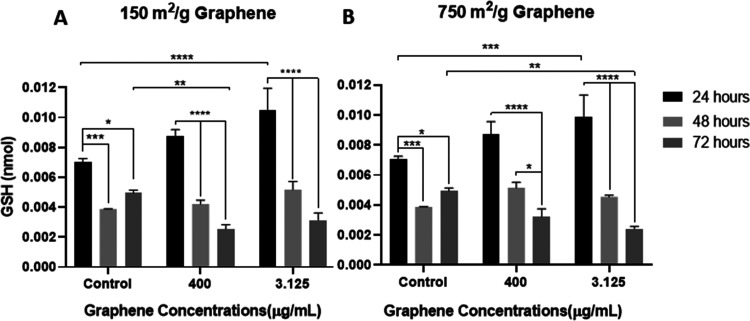
Effects of (A) 150 and (B) 750 m^2^/g graphene in total GSH production. (*p < 0.5,**p ≤ 0.01, ***p 0.001, ****p ≤ 0.0001).

GSH (γ-glutamylcysteinylglycine) is another indicator of oxidative stress in cells.^64^ When the results of the GSH test were examined, an increase in the GSH values was observed, similar to the MDA values, in the cells that encountered both graphene types in the first 24 and 48 h (Figure 4). This increase indicates that graphene does not create oxidative stress on cells; on the contrary, it may have an antioxidant effect.

In many studies in the literature, it has been shown that GO increases the intracellular MDA concentration in a dose-dependent manner.^65−67^ In addition, it has been determined that low-layer graphene is more active than GO, although it has a lower surface area. This is associated with pristine sp2 carbon sites on basal surfaces rather than H-transfer from hydroxyl groups of scavenging activity. With the increase of the surface area, the OH and O_2_ scavenging capacity also increases.^68^

In the study of Wang et al., an increase in MDA concentrations of GO was observed, and they stated that it caused a decrease in GSH concentrations and that this imbalance causes oxidative stress in cells. Oxidative stress may result from an imbalance between prooxidant (MDA) and antioxidant (GSH) compounds.^66^ In our study, MDA and GSH values indicate antioxidant activity and no imbalance was observed.

The antioxidant effect of graphene has been chemically demonstrated.^69,70^ Although the antioxidant effect on cells is very limited in the literature,^71^ it is possible chemically. The antioxidant effect of our results is important data to explain the positive results of graphene in cell proliferation and resistance to different stress sources,^72−74^ which have been shown in many studies. Also, Wang et al. obtained a relationship between antioxidant activity and surface oxygen groups of graphene and demonstrated by the analysis of X-ray photoelectron spectroscopy (XPS), Raman spectroscopy, and Fourier transform infrared (FT-IR) spectroscopy.^75^

### 3.4 Caspase 3/7

**Figure 5:**
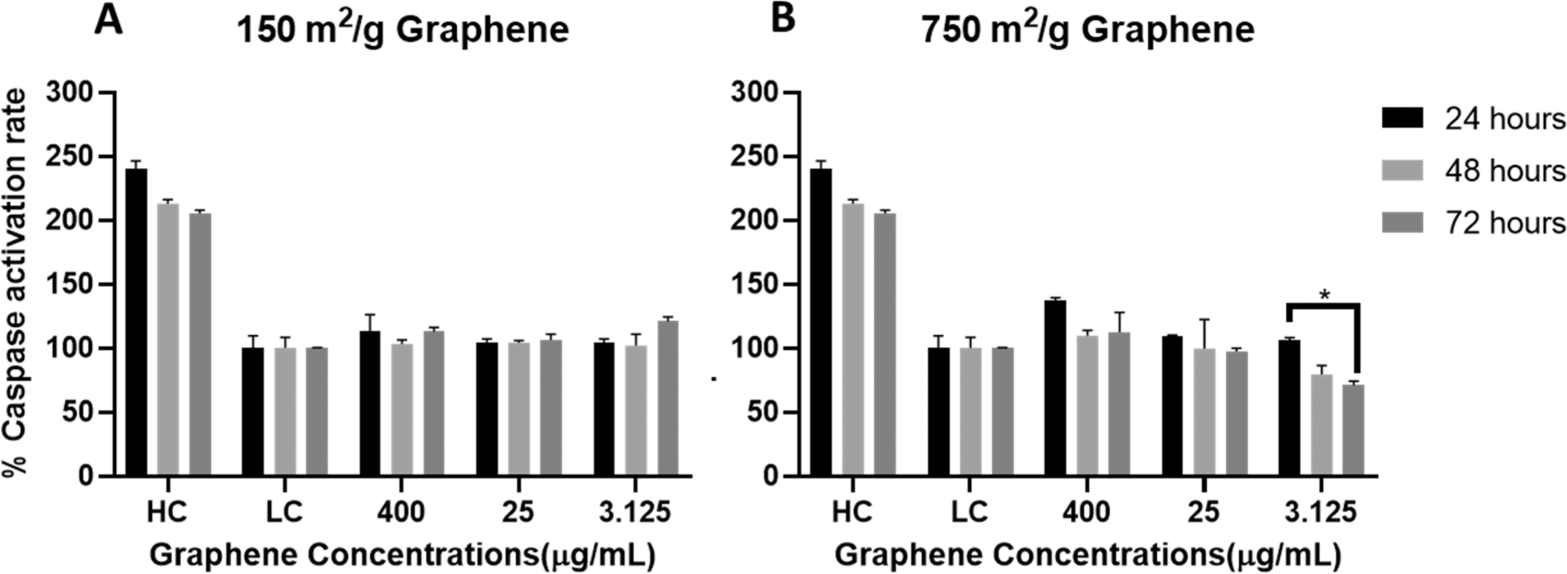
Effects of (A) 150 and (B) 750 m^2^/g graphene in caspase activation rate. No significant change was observed compared to negative control (****p ≤ 0.0001).

Caspase 3 and 7 are mediators of mitochondria-related apoptosis events.^76^Figure 5 shows that cells cultured with graphene have lower caspase activity in both surface areas compared to the control group. Similar results were reported in a study with SH-SY5Y cells cultured with GO, and the cells showed even lower caspase activities compared to the negative control group in lower concentrations, which means GO did not cause apoptosis in SH-SY5Y cells.^77^

In our study, no differences in time-dependent caspase activity were observed, but there are same reports of this in the literature.^67^ While intrinsic or extrinsic pathways can cause caspase activation, obtaining different results in so many graphene-related studies is related to the synthesis methods, sizes, and loads of graphene used in the studies.

Liao et al., in their 2018 review, stated that graphene concentrations ≥10 μg/mL caused apoptosis in Caspase 3 analysis.^61^ Fahmi et al. also stated in their study in 2017 that graphene caused cell death through oxidative damage, and caspase-mediated and caspase-independent pathways.^78^ In our study, on the contrary, Caspase activity was not found at any concentration for the two different surface areas tested.

### 3.5 Genotoxicity

**Figure 6:**
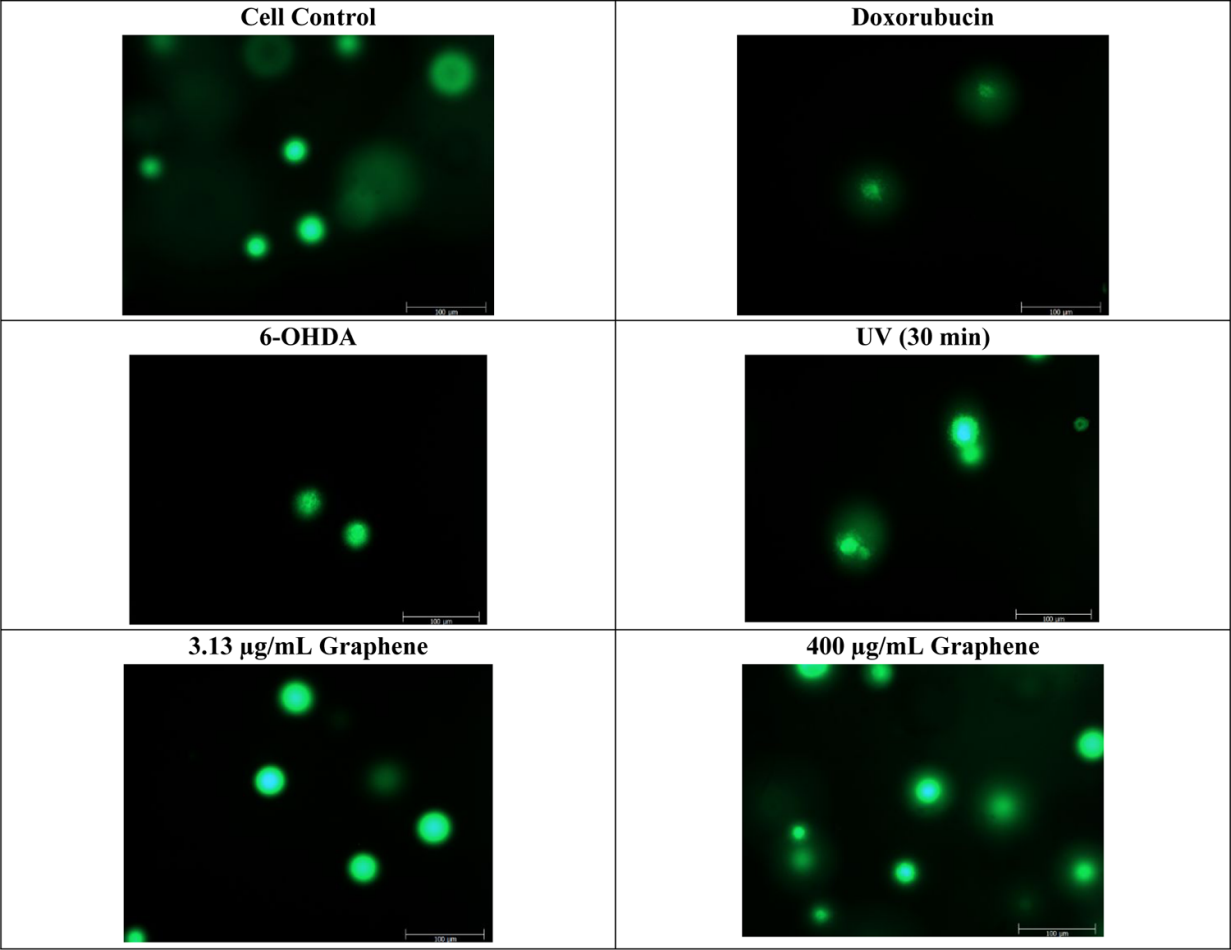
Comet analysis images. Doxorobucin, 6-OHDA, and UV treatment were used as positive controls. Compared to positive controls and negative (cell) control, graphene-treated groups have shown no genotoxic effect. Scale bar shows 100 μm.

Genotoxicity analysis was performed with graphene having a surface area of 750 m^2^/g at 3.13 and 400 μg/mL concentrations. No genotoxic effect was observed for SH-SY5Y cells after 24 h of culture (Figure 6).

Wang et al. showed that GO causes concentration-dependent genotoxicity and as the concentration increases, the genotoxic effect increases.^79^ Akhavan et al. reported that the cyto- and genotoxicities of graphene are highly relevant to the average lateral dimensions. They observed the cell damage with 11 nm average lateral dimensions at low concentrations of GO, while they showed cytotoxicity effect at only high concentrations of 3.8 nm average lateral dimensions.^80^ Qiao et al. examined the genotoxicity of GO-based biomaterials in human fetal fibroblast cells and compared it with the genotoxicity of indium, stannum, silicon dioxide, zinc oxide, and titanium dioxide.^81^ All had dose-dependent genotoxic effects, but graphene caused the most DNA damage. TUNEL analysis demonstrated that pristine graphene induced DNA fragmentation in renal tubular epithelial cells.^78^

On the contrary, it has been shown in our study that graphene has no genotoxic effect; the reason for this may be related to acidity. If GO is synthesized in sulfuric acid, it can have a sulfur content of up to 6%, and the sulfur content is due to covalently bonded sulfate.^82^ The acid content in GO can increase the acidity of the medium and may be expected to cause toxicity.

## 4 Conclusions

Graphene with a surface area of 750 m^2^/g is slightly more toxic than the one with the surface area of 150 m^2^/g. LDH results have concluded that the viability loss of the cells is not through membrane damage. Only the highest concentration of graphene caused membrane damage only at 150 m^2^/g surface area. The results of the MDA test have demonstrated that neither of the two graphene types showed damage through this oxidative stress pathway. The increase in GSH values observed in cells due to both types of graphene, and the decrease of MDA in 750 m^2^/g graphene group suggest that graphene may have an antioxidant effect. This antioxidant effect suggests that graphene has an important potential as a neuroprotective agent, helping resistance to different oxidative stress sources that are associated with several neurodegenerative conditions. In general, the literature reports higher cytotoxicity of GO compared to our results, as well as other limited literature on graphene. This also may be attributed to the antioxidant effect caused by the O_2_ scavenging properties of graphene.

## Notes

TOC is created with BioRender.com.
